# Dynamics of the Ethanolamine Glycerophospholipid Remodeling Network

**DOI:** 10.1371/journal.pone.0050858

**Published:** 2012-12-10

**Authors:** Lu Zhang, Norberto Díaz–Díaz, Kourosh Zarringhalam, Martin Hermansson, Pentti Somerharju, Jeffrey Chuang

**Affiliations:** 1 Department of Biology, Boston College, Chestnut Hill, Massachusetts, United States of America; 2 School of Engineering, Pablo de Olavide University, Seville, Spain; 3 Institute of Biomedicine, Department of Biochemistry and Developmental Biology, University of Helsinki, Helsinki, Finland; University of Georgia, United States of America

## Abstract

Acyl chain remodeling in lipids is a critical biochemical process that plays a central role in disease. However, remodeling remains poorly understood, despite massive increases in lipidomic data. In this work, we determine the dynamic network of ethanolamine glycerophospholipid (PE) remodeling, using data from pulse-chase experiments and a novel bioinformatic network inference approach. The model uses a set of ordinary differential equations based on the assumptions that (1) sn1 and sn2 acyl positions are independently remodeled; (2) remodeling reaction rates are constant over time; and (3) acyl donor concentrations are constant. We use a novel fast and accurate two-step algorithm to automatically infer model parameters and their values. This is the first such method applicable to dynamic phospholipid lipidomic data. Our inference procedure closely fits experimental measurements and shows strong cross-validation across six independent experiments with distinct deuterium-labeled PE precursors, demonstrating the validity of our assumptions. In constrast, fits of randomized data or fits using random model parameters are worse. A key outcome is that we are able to robustly distinguish deacylation and reacylation kinetics of individual acyl chain types at the sn1 and sn2 positions, explaining the established prevalence of saturated and unsaturated chains in the respective positions. The present study thus demonstrates that dynamic acyl chain remodeling processes can be reliably determined from dynamic lipidomic data.

## Introduction

Lipids are fundamental building blocks of cellular membranes and are also essential for signal transduction, energy homeostasis, and many other cellular processes. Recent advances in mass-spectrometry have made large-scale quantification of lipidomes possible [1], [2] and have revealed an unprecedented diversity of lipid species [3]–[5]. Such lipidomics data provide an enormous amount of information, which should eventually lead to understanding of the mechanisms underlying lipid homeostasis and its impact on cellular functions.

Glycerophospholipids are the dominant lipids in mammalian membranes and are comprised of a glycerol moiety, a polar head group linked via a phosphate to the sn3 position of the glycerol moiety as well as an acyl chain esterified to the sn1 and the sn2 positions [6]. The hydrocarbon chain in the sn1-position can also be linked to the glycerol moiety via an alkyl or alkenyl ether bond. Because of these variations as well as the variation of the length and number of double bonds, glycerophospholipids comprise a great number of molecular species. The molecular species composition is regulated by biosynthesis, turnover and acyl chain remodeling (i.e. the Land's cycle [7]), mediated by phospholipases and acyltransferases or transacylases. Distortions of the molecular species distributions can lead to severe pathophysiological consequences and altered lipid distributions have been found in many diseases such as Barth Syndrome, heart failure, type 2 diabetes, and several types of cancer [8]–[12]. Understanding of these distortions is a crucial problem, for cell and developmental biology, potential diagnostics and treatments, and nutrition [13]. The mechanisms by which lipid composition influences human diseases in most cases remain to be elucidated, though lipids have common roles in membrane structure, membrane trafficking, regulation of membrane proteins, and cellular architecture [13]. For example, it was recently shown that obesity increases arachidonic acid in membrane phospholipids, and that subsequent lipid remodeling retargets arachidonic acid to ether lipids. This process is believed to make adipocytes more vulnerable to inflammation [14].

Traditionally, the acyl remodeling process has been studied by addressing the specificity of individual enzymes in vitro using a limited number of substrate species [15]–[18]. The recent advances in lipidomics suggest that deeper understanding could be obtained by applying novel data-mining approaches to lipidomic data, but it remains a challenge to accurately infer the remodeling processes from these complex datasets. Some aspects of acyl remodeling have been revealed by computational approaches. For example, the molecular species composition of cardiolipin can be closely fit by a model in which the four cardiolipin acyl chains are remodeled independently and identically [11], [19], [20]. But the picture is incomplete as the fit breaks down in the case of cancerous tissues and may also be distorted by cell culture conditions [19]. Pulse-chase time course experiments [21], [22] would be superior for determining remodeling mechanisms, but currently there are no computational methods to infer the processes and their associated kinetic parameters from lipid time course data.

In particular, pulse-chase experiments with isotope-labeled precursors (such as choline or ethanolamine) should provide superior information to steadystate measurements [23]–[25], but the interpretation of pulse-chase experiments is complicated by simultaneous labeling of a multitude of molecular species already during the pulse. To avoid this complication, we recently devised a novel approach that allows one to study metabolism of individual phospholipid species in unprecedented detail [21]. A multitude of PE or PS species with a deuterium-labeled head group were synthesized and introduced to cultured cells using cyclodextrin-mediated transfer, and the metabolism of the species in time was monitored by electrospray ionization mass spectrometry. While the PE and PS species similar or identical to endogenous species were hardly remodeled (as expected), those not present endogenously were rapidly remodeled at both the sn1 and sn2 position, eventually yielding a molecular species profile similar to that the endogenous PE and PS. Major differences in remodeling pathways and kinetics were observed between the species within a class, as well as between corresponding PE and PS species. However, due to complexity of the data, the contributions of the alternative remodeling pathways could be only roughly estimated.

To more effectively analyze this type of data, we have now constructed a novel predictive method for determining the lipid remodeling network and its parameter values. We demonstrate the usefulness of this approach by analyzing data from pulse-chase experiments with 6 different exogenous phosphatidylethanolamine precursors, i.e. 14:0-14:0, 14:1-14:1, 18:3-18:3, 18:0-18:1, 18:1-18:1, 18:0-22:6 [21].

## Results

Our model assumes that (1) sn1 and sn2 acyl positions are independently remodeled, (2) remodeling reaction rates do not vary over time and (3) the concentrations of the acyl donors are constant. These assumptions, which are based on previous findings on acyl remodeling of phosphatidylethanolamine, phosphatidylcholine and cardiolipin [19], [20], [26], allowed us to model the system using a simplified framework with a small number of parameters. We have developed a two-step algorithm to automatically determine and solve for key remodeling parameters. This algorithm consists of a flux analysis step to construct and simplify the remodeling network, followed by a B-spline-based parameter inference step that optimally solves the ordinary differential equations governing the system.

**Figure 1:**
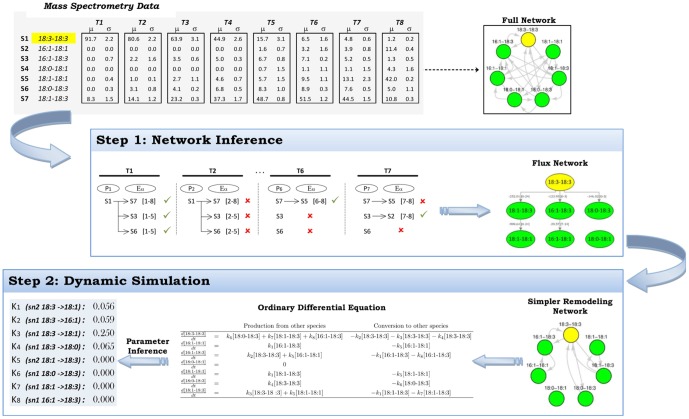
Schematic of the complete inference procedure for determining phospholipid remodeling processes. The procedure is based on two steps using lipid mass spectrometry values: (1) Network inference of the possible acyl remodeling reactions. This yields a simplified remodeling network from the theoretically possible full remodeling network; and (2) Dynamic simulation of the remodeling processes at potential parameter values to fit the lipid mass spectrometry data using the simplified remodeling network. The space of potential parameter values is searched to find the values that best fit the data.

We analyzed a set of 6 separate pulse-chase experiments carried out previously (see Methods and [21]). In those experiments exogenous PE species with a deuterium-labeled head group were introduced to BHK21 cells for 1 hour using a cyclodextrin carrier and the cells were then chased for 24 h. During the chase, a number of new PE species were generated due to extensive acyl remodeling as revealed by ESI-MS analysis. The exogenous PE precursors studied were 14:0-14:0, 14:1-14:1, 18:3-18:3, 18:0-22:6, 18:0-18:1 and 18:1-18:1. The data for each of these experiments were analyzed using a two-step procedure outlined in Figure 1. In this procedure, data from individual pulse chase experiments are used for (1) network inference of acyl remodeling reactions and (2) inference of parameter values for these reactions.

### Inference of the remodeling correlation network

**Figure 2:**
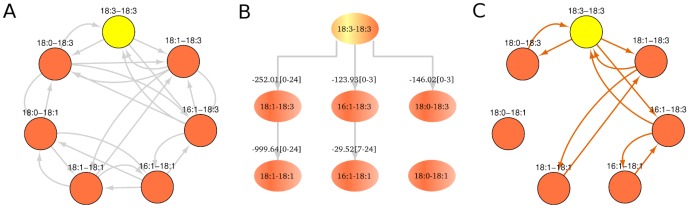
Full PE remodeling network and reduced network based on flux analysis. The full PE remodeling network of 18:3-18:3 pulse-chase experiment consists of all possible acyl chain exchange reactions between lipid species at any time 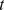, assuming sn1 and sn2 positions are independently remodeled (A). The simpler remodeling network (C) inferred from flux analysis (B) can be adequate for fast and accurate determination of the system. Flux analysis identified reactions with significant evidence that one species converts to the other. A large portion of flux in (B) involves 18:3-18:3 converted to 18:1-18:3 and then converted to 18:1-18:1, according to the edge correlation score (Equation 2) and evidence time range in brackets.

We consider the PE remodeling system as a chemical reaction network, defined by a finite directed graph 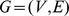, where 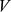 is the set of vertices consisting of PE molecular species, 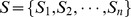, and 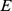 is the set of edges, each representing a remodeling reaction converting source species to target species. Each edge associates with a remodeling reaction rate that depends on the remodeled chain only. Here we use the sn1 and sn2 position independence assumption [26] so that any two connected PE species differ at only one position. Figure 2 (A) shows the full remodeling network for the 18:3-18:3 PE precursor. There can be up to 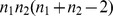 edges in the full network where 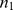 and 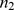 represent the number of sn1 and sn2 chain types, though in practice we do not count edges to PE species that are not experimentally observed as part of the full network. Parameter inference on the full network of interactions would be very slow and is likely to lead to over-fitting.

Remodeling in cells is likely to proceed primarily through a subset of the possible edges in the full network. The first step toward the identification of the dominant remodeling pathways was taken in ref. [21], where the unimportant edges in the full network were eliminated by manually examining the changes in PE species concentrations over time. Here, we extend and automate this process using a fast and accurate correlation network algorithm that reduces the complexity of the full network. Briefly, the algorithm iteratively cycles through candidate “source” and “target” species and examines whether the concentration of the source species is inversely correlated to the sum of the concentrations of the target species. The significance of each connection is calculated using a standard t-test, which can be used to set a threshold for connections. Figure 2 (B) shows the output of the algorithm for the 18:3-18:3 precursor experiment (threshold significance level 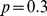) including correlation score and the evidence time interval for each connection. We observed that results are relatively insensitive to the choice of significance level (See Methods).

Figure 2 (C) shows the reduced bidirectional correlation network for the 18:3-18:3 precursor experiment (See Supporting Information S1 for results for the other precursors). As can be seen, a large component of the flux involves the conversion of 18:3-18:3 to 18:1-18:3 and the conversion of the latter to 18:1-18:1. This suggests that remodeling of 18:3 at the sn1 position is faster than at the sn2 position. Similarly, in the 14:1-14:1 precursor experiment, the 14:1-14:1 precursor is first converted at the sn1 position to become 18:1-14:1 and then at the sn2 to become 18:1-18:1 (Supporting Information S1). However, the major path of the 14:0-14:0 experiment differs (Supporting Information S1). 14:0-14:0 is first converted at the sn2 to become 14:0-18:1 and then to 18:1-18:1. An open question is whether the remodeling mechanisms can still be the same in these experiments, and how the apparent differences in sn1 and sn2 ordering can be explained. These points will be addressed below.

### Dynamic simulation

Once a correlation network has been inferred for a given experimental timecourse, the behavior of the system can be forward simulated using ordinary differential equations (ODEs) determined by the reduced bidirectional correlation network. ??Table 1 shows the dynamical system governing the 18:3-18:3 experiment reactions (See Figure 2 (C) for a graph of the network). To infer the values of the parameters in the correlation network, we implemented an expectation-maximization-like B-Spline algorithm in which parameter values are updated iteratively to minimize an objective error function [27] subject to the constraint that the solution satisfies the ODEs that govern the system (See Methods).

**Table 1:** Differential equation model for PE dynamic remodeling.

		Production from other species	Conversion to other species
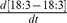	=	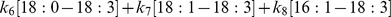	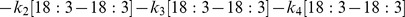
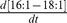	=	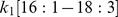	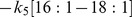
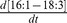	=	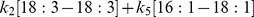	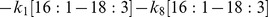
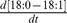	=	0	
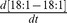	=	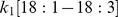	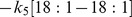
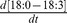	=	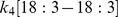	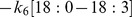
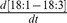	=	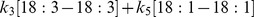	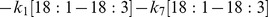

Shown are the ODEs for the 18:3-18:3 remodeling system, according to the correlation network in Figure 2 (C). The reaction parameters 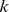 are unknown *a priori*. They depend on the sn1/sn2 position, initial chain type, and product chain type.

**Figure 3:**
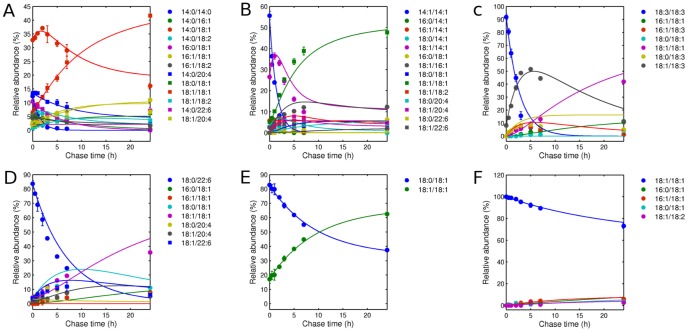
Fit of dynamic simulations for six pulse-chase experiments. Precursors are 14:0-14:0 (A), 14:1-14:1 (B), 18:3-18:3 (C), 18:0-22:6 (D), 18:0-18:1 (E) and 18:1-18:1 (F). Simulations (curve) are in good quantitative agreement with measurements (dots and bars indicate mean and standard error of the mean across replicates). The errors (Equation 8) between prediction and observation are 387, 1064, 2680, 753, 262, and 118 respectively.

**Figure 4:**
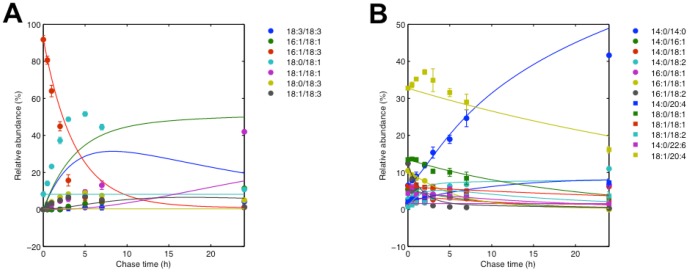
Permutation test result. To examine the robustness of the model, we randomly permuted species labels of the data and used them to solve dynamic system defined by the original data. Shown here are results for the 18:3-18:3 (a) and 14:0-14:0 (b) experiments as examples. As can be seen, the fitting performances are much worse than for the original data, with larger error 5221 compared to original 2680 (a) and 458 compared to original 387 (b). The curves are simulations and the dots are randomly permuted data.

**Figure 5:**
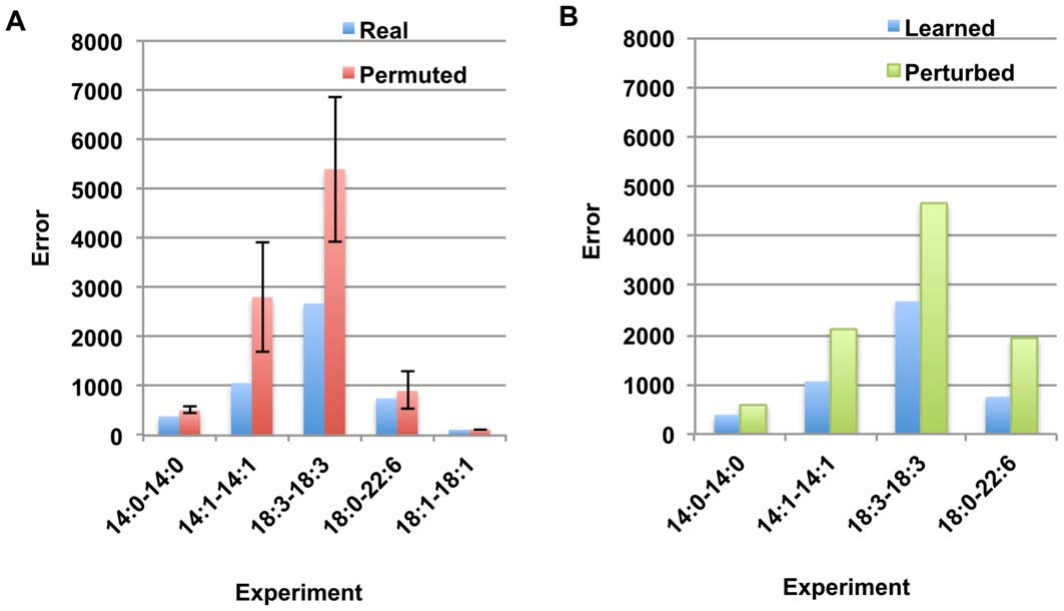
Comparison of fits for the DLipid Procedure on real data to fits for random data or random parameter sets. (Left) Error values for fits to real data (blue) and random label permuted data (red, average and stddev from 100 runs) for 5 separate experimental datasets. (Right) Error values for fits using the parameter set learned in the network inference step of DLipid (blue) and for parameter sets perturbed from this set (green). In the perturbation, parameters for the major reactions sn1 precursor-to-18:1 and sn2 precursor-to-18:1 parameters are deleted and random parameters are added in to replace them, and then inference is performed using this modified parameter set.

Figure 3 shows the fit results for each of the six experiments. For these datasets, we observed that parameter values generally converged within 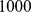 iterations (See Supporting Information S1). As can be seen, the simulation curves closely fit the experimental data, suggesting that the models accurately describe the PE remodeling system. As a control, we repeated the inference procedure on nonsense data generated by permuting labels on the PE species. For the label-permuted data, we saw that fits were systematically worse. Examples of fits for label-permuted data are shown in Figure 4 for the 18:3-18:3 and 14:0-14:0 precursor experiments. We also observed that the inferred parameters displayed much larger variation across iterations in these cases than for the real data (Supporting Information S1). More generally, for each experiment except the 18:0-18:1 precursor experiment we performed random label permuting of the data 100 times and compared the quality of the fit to that found for the real data. The 18:0-18:1 precursor experiment was not used because the original data contains only 2 lipid species. For three of the experiments (14:0-14:0, 14:1-14:1, 18:3-18:3) we observe that the real data is systematically better fit by our procedure than the permuted datasets, as shown in Figure 5 (left). The mean and standard deviation of the error function are shown across 100 permutations for each of the experimental sets. The fit error for the real data is also lower than the average error for the 18:0-22:6 and 18:1-18:1 experiments, though this effect is weaker than for the other experiments.

In addition, we observed that fits were systematically worse when we used parameters other than those that were found in the correlation network inference step. We observed that when we removed the parameters corresponding to the major reactions (sn1 precursor
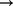
18:1) and (sn2 precursor
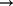
18:1) and added in random parameters to replace them, the fit procedure converged on worse error values. This is shown in Figure 5 (right). These findings demonstrate that the close fits for the real data were not due to overfitting. We note also that it is important to use bidirectional reactions rather than only the 1-way processes observed in the network inference step (See Supporting Information S1). A list of all inferred parameters in all experiments is given in Supporting Information S1.

### Deacylation and reacylation at sn1

Since the same cell line is used in all experiments, we hypothesized that the inferred remodeling parameters should be the same in all experiments, and if so this would support the accuracy of our inference procedure. Primary conversions, i.e. those involving acyl flux from an acyl chain found in the PE precursor, should have the most reliable inferred parameter values. This is due to their high initial abundance in each experiment and the large changes in the precursor concentration over time. Therefore we focused on comparisons of the primary effect parameter values across the experiments. Table 2 shows the primary effect parameter values inferred in each experiment.

**Table 2:** Remodeling rate parameters from six independent experiments.

Experiment precursor	new sn1 chain					new sn2 chain				
	Initial chain	*18:1*	*18:0*	*16:1*	*16:0*	Initial chain	*18:1*	*18:2*	*16:1*	*20:4*
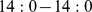	*14:0*	0.0889	0.0166	0.0261	-	*14:0*	0.5176	0.1520	-	-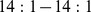	*14:1*	0.5903	0.1083	-	-	*14:1*	0.1400	0.0998	0.1153	0.0769
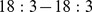	*18:3*	0.2500	0.0650	0.0589	-	*18:3*	0.0555	-	-	-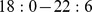	*18:0*	0.0673	-	-	-	*22:6*	0.0724	-	-	0.1146
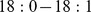	*18:0*	0.0700	-	-	-	*18:1*	-	-	-	-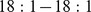	*18:1*	-	0.0019	0.0072	0.0041	*18:1*	-	0.0029	-	-

Inferred remodeling rate parameters from six independent experiments. A “-” indicates unmodeled reactions either due to missing species or low flux in the experiment. The sn1 18:0 to 18:1 rate parameters inferred from two precursor experiments are similar: 0.0673 and 0.07 for the respective 18:0-18:1 and 18:0-22:6 experiments. The sn1 rate parameters of the first row have ratios 1:0.19:029, similar to ratios in the second row 1:0.18:- and third row 1:0.26:0.24. This indicates that the relative reacylation rates of 18:1, 18:0, and 16:1 are consistently ranked across experiments. Likewise, by comparing values within a column, we can determine relative deacylation rates, i.e. 14:1
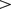
18:3
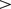
14:0 at ratios 6.58:3:1 at the sn1 position. Analogous behavior was also observed for the sn2 position (See Supporting Information S1 for details).

Remarkably, the individually solved remodeling mechanisms from the six experiments show strong consistencies, suggesting that our model correctly describes the behavior of the biological system. For example, the sn1 18:0 to 18:1 conversion rates inferred from the two precursor experiments (18:0-22:6 and 18:0-18:1) are consistent (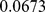 and 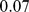 respectively). While conversion rates were dependent on the initial chain and the new chain, we observed certain regularities. For example, chains convert at varying rates to 18:1 at the sn1 position. The fastest converting chain is 14:1 and the slowest is 18:0, with overall order 14:1
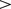
18:3
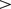
14:0
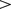
18:0 (relative rates 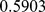, 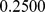, 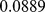, 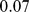). Since all of these chains are being converted to the same product, rates must differ because of differences in deacylation rate. 14:1 is the most rapidly deacylated sn1 chain while 18:0 is the one most slowly deacylated. Analogously, when we examined the rates associated with conversion to 18:0 at the sn1 position, we saw that 14:1 deacylated the fastest, just as we saw that 14:1 deacylated the fastest in conversions to 18:1. In fact, the 18:1 column and the 18:0 column both have (14:1, 18:3, and 14:0) as their three fastest deacylating chain types. This suggests that the same deacylation processes are active in all experiments and that our method is accurately detecting them.

We observed a similar effect with reacylation. To determine whether the reacylation rate is also dependent on the chain type, we examined the relative rates of sn1 chain conversion to 18:1, 18:0 and 16:1. For the 14:0-14:0, 14:1-14:1, and 18:3-18:3 PE precursors, the ratios of the conversion rates of the sn1 precursor to 18:1, 18:0 and 16:1 were 1 : 0.1 : 0.29, 1 : 0.18 : (no data), and 1 : 0.26 : 0.24, respectively. Thus these ratios are relatively robust. (Normalization by the precursor-to-18:1 rate is necessary to account for differences in the deacylation rate of the precursors) This behavior indicates that common mechanisms are active in separate precursor experiments. Therefore, cross-validation using independent precursors should be generally effective for determining model robustness. The observed consistency of rates also indicates the accuracy of our methodology.

More generally, we were able to distinguish deacylation and reacylation rates and determine relative rates for different chain types by integrative analysis of the combined data using the steady state approximation for the concentrations of reaction intermediates (See Methods). For example, by normalizing the fitted sn1 kinetic parameters in Table 2 to 18:1, which is the fastest converted chain type, we obtain that on average, the sn1 reacylation rates have the order 18:1
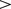
16:1
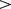
18:0 (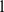 : 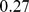 : 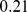 – see Supporting Information S1). Reacylation rates are controlled by the abundance of the given chain type in the donor lipid pool, and selectivity is determined by the acyltransferases/transacylases involved. We observe that the deacylation rates decrease in the order 14:1
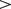
18:3
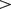
14:0
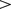
18:0
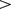
18:1 (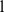 : 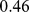 : 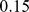 : 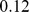 : 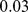 – see Supporting Information S1), reflecting the specificity of phospholipase A1. Thus unsaturated chains are removed faster from the sn1 position than saturated ones, which at least in part explains why saturated chain types are enriched at sn1. Consistent with our previous suggestion [21], removal of 18:1 from the sn1 position is considerably slower than that of other acyls such as 14:0 and 18:3. For more detail on calculation of deacylation and reacylation rates, see Method and Supporting Information S1.

### Deacylation and reacylation at sn2

Robust deacylation and reacylation rates were also found for the sn2 position, although there were less data than for the sn1 position. For example, the relative conversion rate of 14:0 to 18:1 vs. 18:2 was 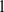 : 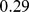 (See Table 2 and Methods). This ordering is in agreement with the conversion rates of precursors having 14:1 at the sn2, i.e. conversion to 18:1 was preferred to 18:2 in a ratio of 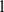: 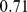. Based on the integrative analysis described above, we determined that the deacylation rates of different chain types decrease in the order 14:0
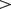
14:1
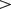
22:6
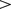
18:3 (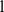 : 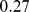 : 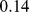 : 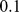). It is interesting to note that the saturated or monounsaturated chains are removed faster than polyunsaturated ones, thus providing a possible explanation why the sn2 position is enriched in polyunsaturated acyl chains. Removal of 18:1 from the sn2 position also appears to be slower than that of 14:0 and 14:1. An unexpected observation is that the sn2 22:6 chain of 18:0-22:6 PE precursor converts to 20:4 much faster than to 18:1. This conflicts with the reacylation rates predicted from the behavior of a 14:1 chain at sn2, which is replaced by 18:1 twice as fast as by 20:4 in the 14:1-14:1 precursor. This phenomenon is worth further investigation as it may indicate a cooperative interaction between sn1 and sn2 chain remodeling.

We also observed consistencies in secondary effect parameters. For instance, the sn1 16:1 to 18:1 conversion rate was found to be 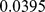 for the 14:0-14:0 precursor and 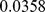 for the 18:1-18:1 precursor. Also, the sn2 18:2 to 18:1 conversion rates were determined to be 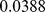 for the 14:0-14:0 precursor and 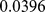 for the 18:1-18:1 precursor. Since these inferences are based on conversions from lower abundance acyl species not originating in any precursor, they may be more sensitive to experimental errors. Future experiments with precursors having sn1 16:1 or sn2 18:2 would be beneficial to determine the accuracy of the approach for secondary rate parameters.

## Discussion

Due to the complexity of lipid remodeling and the lack of previous computational tools, quantitative interpretations of lipidomic data have been rare [1]. In this work, we have presented the first method for inferring the processes and kinetic parameters of phospholipid remodeling from lipidomic data. This method required two steps: network structure inference using a fast correlation analysis step, and inference of kinetic parameters through an efficient B-Spline based optimization approach for fitting the dynamical system. This work provides a significant advance compared to previous lipidomic data mining approaches for acyl chain remodeling, which focused on steady-state systems [19] or were based on dynamic simulations without an explicit method for parameter inference [20]. These acyl chain remodeling studies are complementary to works addressing metabolic fluxes between classes of lipids with different head groups [28], [29].

By applying our new method, we have revealed a number of important facts about lipid remodeling. We have shown that 18:1 is the predominant reacylated acyl chain at both sn1 and sn2 positions, consistent with previous findings [21]. We were also able to determine the deacylation and reacylation rates of different types of acyl chains. For example, we found that unsaturated chains are cleaved off more rapidly than saturated ones at the sn1 position (14:1
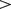
18:3
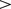
14:0
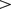
18:0), while at the sn2 position saturated chains are more quickly removed (14:0
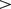
14:1). These data explain why saturated acyl chains are predominant in the sn1 position and unsaturated chains are predominant in the sn2 position of diacyl glycerophospholipids.

**Figure 6:**
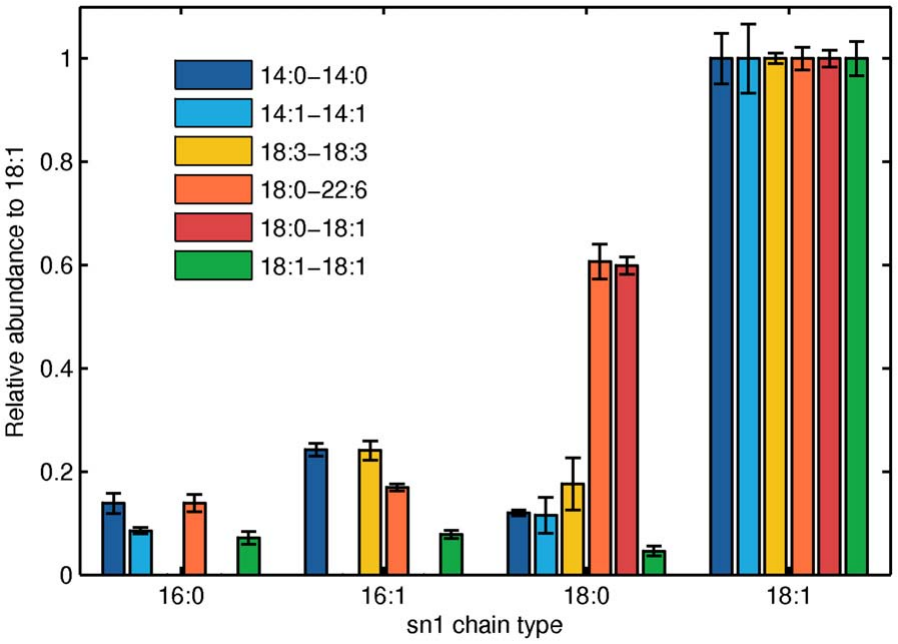
The distribution of acyl chains at the sn1 position at the 24 hr timepoint in each experiment, normalized by the amount of 18:1 at the sn1 position. For further details and comparable sn2 data, see Supporting Information S1.

To appreciate the importance of the computational modeling approach we have developed, it is useful to compare the results with those that would have been deduced by empirical observation of the data. Figure 6 shows the distribution of acyl chains at the sn1 position of PE at the final time point in each experiment, normalized by the amount of 18:1 at the sn1 position (See also Supporting Information S1). This is when the experiments have had the most time to equilibrate, so if similar remodeling processes are occurring in each experiment we would expect the acyl distributions to be similar. However, we observe that there are still substantial residual effects from the precursors. The most salient effects are that there are much larger amounts of 18:0 at the sn1 position in the 18:0-22:6 and 18:0-18:1 precursor experiments than in the other precursor experiments. Under purely empirical interpretation, this precursor bias might lead one to believe that remodeling processes differ across experiments. However, our quantitative modeling approach allowed us to determine that remodeling processes are in fact consistent across experiments. It is remarkable that our simulations not only all provide good fits but also cross validate in the parameter values, despite the fact that mass spectrometry measurements used to obtain the raw data have some positional isomer and missing data uncertainties. This suggests that the model is not excessively sensitive to measurement uncertainties and thus captures the essential features of the remodeling process in vivo.

Empirical interpretation also has other shortcomings. For example, while the prior empirical interpretation suggested several interactions between sn1 and sn2 chains [21], our modeling approach indicates that the data can be well fit even if sn1 and sn2 chains react independently. Our modeling approach also has the advantage of providing quantitative estimates of kinetic parameters, which are non-obvious in empirical analysis. For example, we were able to determine that the sn1 deacylation rate varies by an order of magnitude depending on the type of acyl chain.

A strength of our method is that it is based on the straightforward position-independence assumption [26], which has previously found to be valid in most contexts for the tetra-acyl phospholipid cardiolipin [19]. The success of the present model indicates sn-position independence is typical for glycerophospholipid acyl chain remodeling. While certainly models involving large number of parameters and more cooperative effects should be able to fit the data, we have focused on an independence model due to the standard criterion of wishing to keep the number of parameters to a minimum. However, we do note that occasional deviations from independence were observed in this study, e.g. in the analysis of sn2 reacylation behaviors, suggesting that in a few cases interactions among the acyl chains may influence the remodeling process. This interaction/cooperativity could relate to overall molecular hydrophobicity, which has been recently strongly implicated in the specificity of A-type phospholipases [30].

In summary, we have constructed a powerful modeling tool for the analysis of glycerophospholipid remodeling pathways and their kinetics. The present work demonstrates that computational methods can quantitatively determine the details of glycerophospholipid remodeling by identifying the specificity and kinetics of deacylation and reacylation. Notably, the present method can be readily extended to other glycerophospholipid classes and should thus allow one to obtain a comprehensive picture of lipid remodeling, which will be essential for understanding lipid homeostasis in mammalian cells. A natural future application will be determining the mechanistic impact of various types of perturbations, e.g. knockdown of putative remodeling enzymes. Software implementations for the Correlation Network (JAVA) and Dynamic Simulation (MATLAB) are available at http://nbidiaz.github.com/DLipid/.

## Methods

### Network Inference

The remodeling pathway of phospholipids can be thought of as a directed weighted graph 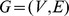 where the nodes represent the lipid species 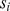, 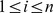, and the directed edges 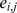, 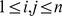 indicates the flux. Here 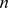 denotes the total number of species. The weight of each edge is proportional to the degree of the flux from source species to the target species. Here we describe a novel algorithm for inferring such a network from MS time course measurement such as in pulse chase experiments. Let 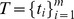 be the measurement times, and 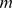 is the total number of measurements. At each time point 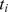 all species whose concentration levels are decreasing in the next time point, at a significance level 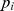 as determined by t-test, are identified as possible remodeling sources. By a source we mean a species that is remodeled into a target species. The significance level 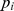 depends on the time point and is defined by 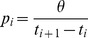. In practice, parameter inferences are relatively insensitive to the choice of significance level. An example of this is shown in Table 3.

**Table 3:** The primary parameter values of 14:0-14:0 experiment are stable using different correlation network thresholds.

threshold	num edges	num para	error	sn1 14:0 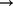 18:1	sn1 14:0 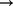 16:1	sn1 14:0 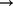 18:0	sn2 14:0 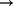 18:1	sn2 14:0 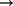 18:2
0.1	16	14	400	0.0893	0.0312	-	0.6197	-
0.2	26	22	394	0.0911	0.0256	0.0213	0.5260	0.1216
0.3	32	24	387	0.0889	0.0261	0.0166	0.5176	0.1520
0.4	50	38	372	0.0801	0.0269	0.0130	0.3836	0.1062
full	66	50	371	0.0688	0.0242	0.0163	0.3426	0.1018

Each row indicates the number of edges, number of parameters, fit error, and inferred parameter values at a given significance threshold for determining edges in the correlation network.

Denote the set of candidate sources at the 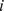
th time point by 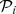. For each 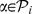, we define the neighborhood 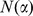, to be the set of lipid species 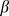 that share exactly one chain with 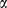. Next we introduce a correlation function that helps to determine whether 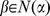 could be the remodeling product of 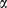. For a lipid species 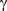, define
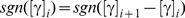
(1)where the 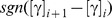 is determined according to the t-test at the significance level 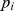. Let
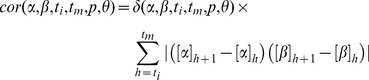
(2)where 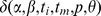 is a sign function that is negative if 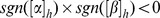 for all 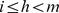 and positive otherwise. This 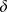 function is introduced to capture the inverse relation between the concentrations of 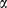 and 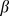 over the entire time interval 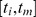. Note that 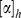 and 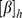 denote the concentration of 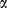 and 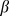 species at *h*th time point, respectively.

For each candidate source 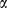, we associate a set 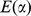 of edges. The set 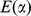 is initialized to the empty set. We then cycle through candidate target set 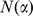, and for each member 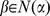, we add an edge 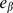 to 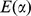 if 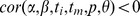 or if there exist a species 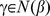 such that 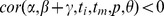. The second correlation is introduced to account for the possibility that 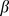 is remodeled to 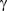 which reduces the concentration of 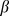. 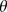 is defined as the significance threshold value in Algorithm 1 (Supporting Information S1). We select the 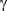 with highest correlation value. Since the concentrations of species vary in time, some fluxes may only be visible at later time intervals. These new fluxes may influence the correlations at the earlier time points. For this reason, If no negative correlation is found in the time interval 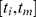, the final time point 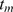 is reduced to the previous time point 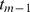 and the whole process is repeated. We do this until an edge is found or we reach 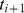. These recursions are repeated for the entire source set. Lastly, the weights of the edges are set to the correlation values. See SI for the pseudo-code of this algorithm.

### Dynamic Simulations

The remodeling process of PE is a dynamical system that can be modeled by a set of coupled differential equations. This dynamical system depends on the rates of conversion of PE species into one another. These rates are not known a priori. Denote these parameters by the vector 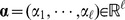, where 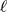 denotes the total number of parameters. Let 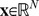 represent the vector of PE concentrations. Here 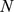 denotes the total number of species. The dynamical system describing the PE remodeling is then
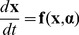
(3)Given a set of observations 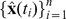, there are a number of available numerical approximations that can be used to approximate the parameters 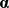 using the observed time-course measurements, while simultaneously solving the differential equations [31]–[33]. Since the dependence of the vector field  on the parameters is linear (See Table 1 for an example), we may rewrite the parameter inference problem as a minimization problem that can be efficiently solved using *Singular Value Decomposition, SVD*, or *QR* decomposition as described in [27]. We write the solution 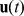 of the above system as a linear combination of cubic B-splines,
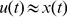
(4)

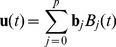
(5)where 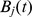 are the B-splines and 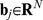 are the spline coefficients [34], [35]. Define the error function 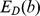 as the square of the distance of the solution 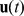 to the observation points as follows.
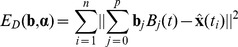
(6)Our goal is to minimize the above error function, while simultaneously ensuring that the solution satisfies the differential equations. The constraint that 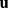 has to satisfy the differential equations second constraint can also be stated in terms of a minimization problem. More precisely, if we have estimates 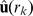 of the solution at the so called “collocation points”, 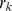, we can define the error function
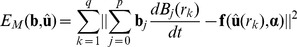
(7)which yields the overall error function
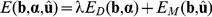
(8)where 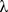 is a weight parameter that controls the weight given to the observations versus the differential equations. Note that the since the dependence of 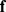 on 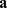 is linear, 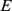 is linear in 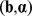. This minimization problem is then readily solved using SVD or QR decomposition. In practice, the approximations 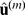 are fitted in an iterative manner where at the 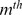 step, 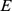 is minimized with respect to 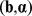 and the resulting 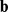 is used to generate the new estimate 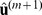. The initial estimate 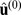 is set to the solution of the minimization of Equation 6. In our calculations the 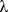 parameter was set to 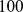. 5 B-splines were used for initial shooting. 21 B-splines and 49 (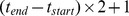) collocation points were used for precise approximation to the real function. We iterated for at least 1000 steps and then stopped at the solution which gives the minimum error among the last 6 steps, to correct for periodicity issues. The algorithm also stops when the error changes too slowly, i.e. 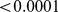 between two subsequent steps. Improvement of the fit from the initial guess was in general large and occurred rapidly in the first few steps of the iterative process (Supporting Information S1). Some parameters exhibited periodicity in the convergence process, which may be related to the shape of the solution space. In such situations, we only tracked the solution set which gives the lowest error. These other periodic solutions yielded parameter values which were similar to the solution with the lowest error (Supporting Information S1).

As an additional verification, the inferred parameters were used to independently solve the differential equation using standard numerical techniques. In our implementation we used the matlab function ode45 with stepsize 0.01 with initial conditions and remodeling rates as solved by the dynamic algorithm.

### Deacylation and Reacylation Rates

Deacylation and reacylation rates were calculated from the 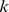 values given in Table 2. Note that in Table 2 the conversion rates 14:0
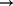
18:1 and 14:0
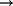
18:0 have a ratio of 5.36, which is relatively similar to the ratio of the rates 14:1
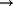
18:1 and 14:1
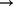
18:0 (5.45) and the ratio of 18:3
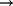
18:1 and 18:3
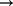
18:0 (3.85). This suggests that new acyl chains are added onto lysophospholipids by a similar process in all experiments. One possible explanation for this is that the pool of available acyl chains is similar in all experiments, which is reasonable since all the experiments were run under the same conditions and because the acyl distribution becomes increasingly similar in all experiments over time [21]. Assuming that chain-specific reacylation processes are similar in different precursor experiments, absolute conversion rates therefore differ across experiments because of differences in chain-specific deacylation rates. Based on this logic, relative deacylation rates can be determined from the ratio of values between rows, while relative reacylation rates can be determined from the ratios of values between columns. For each comparison, deacylation and reacylation rate ratios were calculated via an average using the rates found to exist in the inferred network.

The relative ratios between rows and columns can be formally shown to indicate relative deacylation and reacylation rates by consideration of the kinetics of all species and deacylated intermediates using the steady state approximation for the intermediates. This approximation is justified by the fact that fully acylated PE is more prevalent than lyso- species in typical cells. Since the sn1 and sn2 positions are generally independent, we can consider the behavior of the sn1 (or sn2) position alone. For each chain type 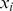 at the sn1 position, the kinetics of 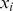 are given by 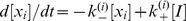, where 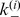 is the deacylation rate of 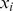, 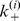 is the reacylation rate of 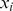 and 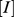 is the concentration of the deacylated intermediate. Assuming that all deacylations lead to the intermediate 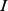 and that 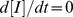, solution of the set of linear equations yields that the rate parameter in Table 2 from initial chain 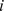 to new chain 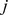 is 
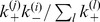
. From this it follows that the ratios of rows and columns indicate relative deacylation and reacylation rates. For example, for relative reacylation rates of chain types 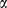 and 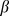 we have 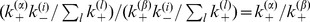. For relative deacylation rates of chain types 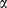 and 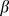 we have 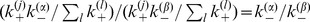.

### Mass Spectrometry Data Analysis

The kinetics of remodeling were derived from data obtained by using neutral loss scanning which allows selective detection of labeled vs. unlabeled PE molecules, but does not provide definitive information on the acyl substituents or their sn-positions. However, such information was obtained in separate experiments in which the cellular PE molecules were fragmented using collisionally-activated decomposition and the products were analyzed as described in [21]. Several studies have shown that the identification of the acyl substituents and the assignment of their sn-positions in glycerophospholipids can be obtained by using such an approach [36]–[38]. Our own studies with several pairs of PE positional isomers yielded three main types of product ions: i) fatty acid carboxylate anions, ii) lysoPL formed upon neutral loss of a fatty acid residue as a ketene and iii) a lysoPL-like lipid due to neutral loss of a free fatty acid. Tests with many pairs of synthetic PE isomers with a saturated and an unsaturated fatty acyl residue (e.g. 16:1/18:1-PE and 18:1/16:1-PE) showed that the relative peaks areas of the carboxylate anion and lysoPE fragment change in a predicated manner with the isomer ratio (Hermansson and Somerharju, unpublished data). Based on those data, we were able to decompose the contributions of the sn1/sn2 isomers in each experimental sample by linear fitting. We estimate that the positional isomers in the cellular PE species can be quantified with an error less than 10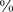.

## Supporting Information

Supporting Information S1 Supporting information.
(PDF)
