# Supplementary material for: Dynamics of the Ethanolamine Glycerophospholipid Remodeling Network
Source: PLoS One. 2012 Dec 10;7(12):e50858. doi: 10.1371/journal.pone.0050858 (PMC3519547; doi:10.1371/journal.pone.0050858)
Supplement: Supporting Information S1 — Supporting information. (PDF) [file pone.0050858.s001.pdf]

Supporting Information
Dynamics of the ethanolamine glycerophospholipid lipid remodeling network

Lu Zhang[1,†], Norberto Díaz–Díaz[2,†], Kourosh Zarringhalam[1,†], Martin Hermansson[3], Pentti Somerharju[3], Jeffrey Chuang[1,*]

1 Department of Biology, Boston College, Chestnut Hill, MA

2 School of Engineering, Pablo de Olavide University, Seville, Spain

3 Institute of Biomedicine, Department of Biochemistry and Developmental Biology, University of Helsinki, Helsinki 00014, Finland

† These authors contributed equally.

∗ E-mail: chuangj@bc.edu

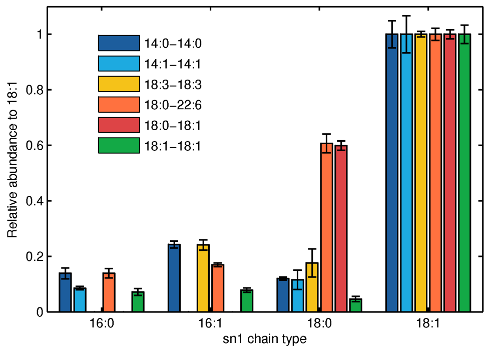

(a)

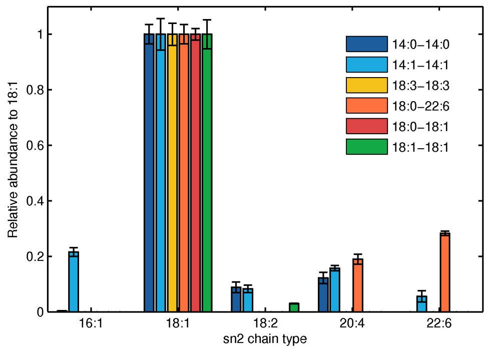

(b)

Figure 1: PE acyl chain proportion at the final time point after chase. The set of allowed chains at the sn1 and sn2 positions are $i \in \Omega_{sn1}, \Omega_{sn1} = \{14{:}0,14{:}1,18{:}3,16{:}0,16{:}1,18{:}0,18{:}1\}$ and $j \in \Omega_{sn2}, \Omega_{sn2} = \{14{:}0,14{:}1,18{:}3,16{:}1,18{:}1,18{:}2,20{:}4,22{:}6\}$, which are the collections of observed chain types among the six experiments. The abundances of chain types at sn1(a) and sn2 (b) were normalized by the predominant reacylated chain type (18:1) to control for the total amount of reacylation. This normalization allows us to compare newly generated chain types, distinguish precursor effect and detect missing species. We ignored chain types that appear to arise only from their experimental precursors. As can be seen, ignoring species with missing data, the distribution of acyl abundances is relatively similar across experiments. At the sn1 position, the 16:0 abundance is consistent (all values 0.12±0.03) for precursors 14:0-14:0, 14:1-14:1, and 18:0-22:6. Likewise, the 16:1 abundance is consistent between experiments 14:0-14:0, 18:3-18:3, 18:0-22:6 (0.22±0.04). 18:0 is consistent between experiments 14:0-14:0, 14:1-14:1, and 18:3-18:3 (0.14±0.03) and between two experiments as precursor 18:0-18:1 and 18:0-22:6 (0.60 and 0.61). However, non-18:1 abundances for the 18:1-18:1 precursor experiment are systematically depressed, likely due to bias from the precursor. At the sn2 position, 14:0-14:0 and 14:1-14:1 experiments have consistent proportions of 18:2 (0.086±0.004) and 20:4 (0.14±0.02). However, there seem to an absence of species containing those chain types in the 18:3-18:3, 18:0-18:1 and 18:1-18:1 experiments. In fact, in the 18:3-18:3 experiment, labeled species concentrations only sum up to 76%. We also observed that the sn1 16:1 chain type is underestimated in the 14:1-14:1 experiment and more abundant at the sn2 position than that from 14:0-14:0, which may be due to uncertainty in isomer identification. However, despite these discrepancies, the data largely agree on their remodeled chain type proportions.

Algorithm 1 (Network Inference pseudocode)

INPUT
  $\mathcal{S} = \{s_1, \ldots, s_n\}$: *lipid species*
  $\mathcal{T} = \{t_1, \ldots, t_m\}$: *time points*
  θ: *significance threshold value*
OUTPUT
  G=(V,E): Remodeling pathway
begin
  $V := \mathcal{S}$
  for $i := 1 \rightarrow (m-1)$ ***do***
    for all *species* $\alpha \in \mathcal{P}_{t_i}$ ***do***
      $< E(\alpha), f > := < \emptyset, m >$
      while $E(\alpha) = \emptyset$ *AND* $f > i + 1$ ***do***
        for all *specie* $\beta \in N(\alpha)$ ***do***
          if $cor(\alpha, \beta, t_i, t_f, \theta) < 0$ *OR*
          $(cor(\alpha, \beta + \gamma, t_i, t_f, \theta) < 0$ *AND* $\gamma \in N(\beta))$ ***then***
            $E(\alpha) := E(\alpha) \cup \{e_\beta\}$
          end if
        end for
        $f := f + 1$
      end while
      $E := E \cup E\prime$
    end for
  end for
end

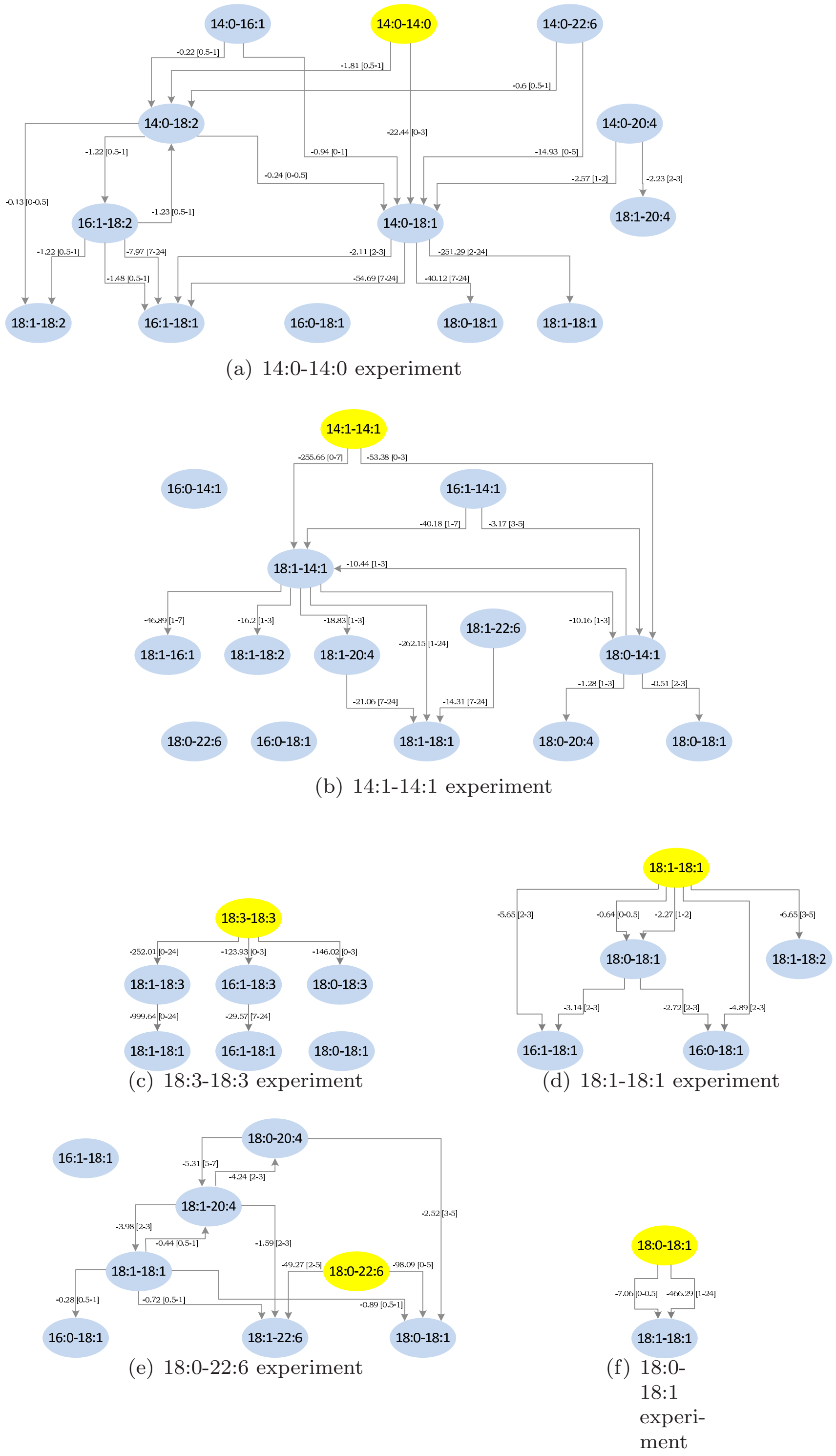

(a) 14:0-14:0 experiment

(b) 14:1-14:1 experiment

(c) 18:3-18:3 experiment

(d) 18:1-18:1 experiment

(e) 18:0-22:6 experiment

(f) 18:0-18:1 experiment

Figure 2: Flux analysis results of pulse-chase experiments with precursor 14:0-14:0 (a), 14:1-14:1 (b), 18:3-18:3 (c), 18:1-18:1 (d), 18:0-22:6 (e) and 18:0-18:1 (f). Edge labels indicate significance score and evidence time range in brackets. All experiments display edges passing algorithm threshold 0.3. As can be seen in (a), 14:0-14:0 is first converted to 14:0-18:1 and then to 18:1-18:1, indicating the precursor's sn2 position is remodeled faster than sn1. However, a large component of the flux in (c) involves 18:3-18:3 first converted to 18:1-18:3 and then converted to 18:1-18:1. Similarly, 14:1-14:1 (b) is first converted to 18:1-14:1 and then converted to 18:1-18:1. (b) and (c) suggest their precursors' sn1 position is remodeled faster than sn2 position. Note that species without any arrows connecting them to other species are those found in the experimental data but without significant correlations. Some species may be connected twice to another species, indicating significant correlations at two disjoint intervals.

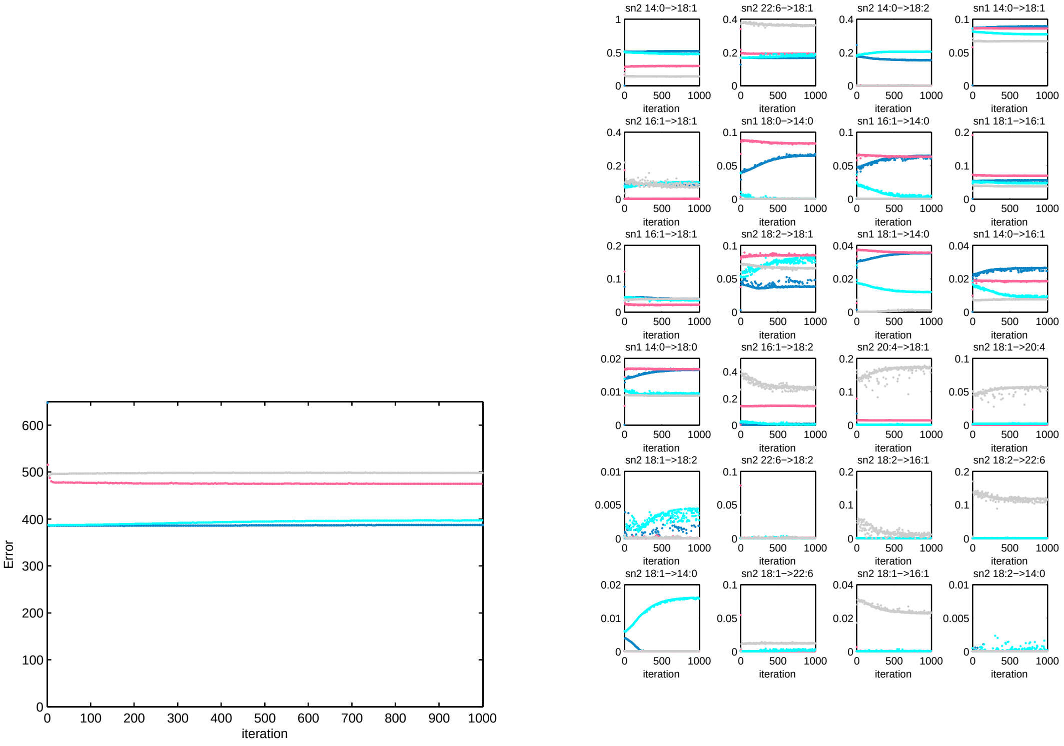

(a) (b)

Figure 3: Parameter inference of 14:0-14:0 experiment using B-Spline algorithm. Shown are the error (a) and parameter values (b) in 1001 iterations. Parameter values display 4-fold periodicity, shown in different colors: blue, pink, cyan, gray. This may be related to the shape of solution space. The blue curve solution gives the closest fit to measurement with lowest error.

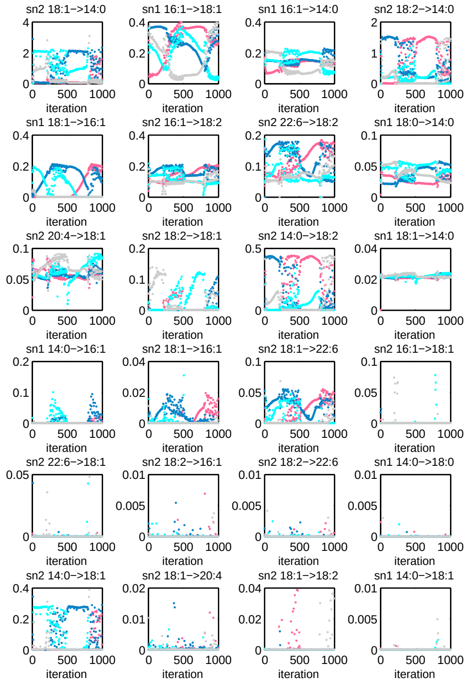

Figure 4: Predicted parameter values using randomly permuted 14:0-14:0 data do not converge.

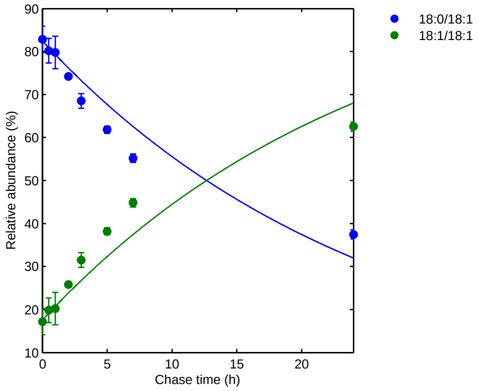

Figure 5: Bidirectional reactions are important for accurately solving the system. If we only use directional reactions as found in the correlation network inference step, we obtain poor fits to the data. For example, if we only include the 18:0→18:1 reaction as shown here, a much poorer fit is obtained than when the reaction in the opposite direction (18:1→18:0) is added, as shown in Figure 3E.

Table 1: Summary of parameter inference results. Zero value parameters are not shown. $*$ indicate the primary effect parameters.

**Experiments**	**sn1 parameters**		**sn2 parameters**	
**14 : 0 − 14 : 0**				
	14:0 → 18:1*	0.0889	14:0 → 18:1*	0.5176
	18:0 → 14:0	0.0652	22:6 → 18:1	0.1669
	16:1 → 14:0	0.0638	14:0 → 18:2*	0.1520
	18:1 → 16:1	0.0561	16:1 → 18:1	0.0808
	16:1 → 18:1	0.0395	18:2 → 18:1	0.0388
	18:1 → 14:0	0.0354	16:1 → 18:2	0.0100
	14:0 → 16:1*	0.0261	20:4 → 18:1	0.0005
	14:0 → 18:0*	0.0166		
**14 : 1 − 14 : 1**				
	14:1 → 18:1*	0.5903	20:4 → 18:1	0.2832
	14:1 → 18:0*	0.1083	18:2 → 14:1	0.2744
			22:6 → 18:1	0.1564
			14:1 → 18:1*	0.1400
			16:1 → 14:1	0.1337
			14:1 → 16:1*	0.1153
			14:1 → 18:2*	0.0998
			14:1 → 20:4*	0.0769
			18:1 → 14:1	0.0458
			18:1 → 22:6	0.0067
**18 : 3 − 18 : 3**				
	18:3 → 18:1*	0.2500	18:3 → 18:1*	0.0555
	18:3 → 18:0*	0.0650		
	18:3 → 16:1*	0.0589		
**18 : 0 − 22 : 6**				
	16:0 → 18:1	0.4093	20:4 → 22:6	0.1253
	18:1 → 16:0	0.0869	22:6 → 20:4*	0.1146
	18:0 → 18:1*	0.0673	22:6 → 18:1*	0.0724
	18:1 → 18:0	0.0067	18:1 → 22:6	0.0036
**18 : 0 − 18 : 1**				
	18:0 → 18:1*	0.0700		
	18:1 → 18:0	0.0346		
**18 : 1 − 18 : 1**				
	18:0 → 16:0	0.0373	18:2 → 18:1	0.0396
	16:1 → 18:1	0.0358	18:1 → 18:2*	0.0029
	16:0 → 18:1	0.0338		
	16:1 → 18:0	0.0283		
	18:1 → 16:1*	0.0072		
	18:1 → 16:0*	0.0041		
	18:1 → 18:0*	0.0019		

Table 2: sn1 relative deacylation rates. Rates in each row have been normalized by the conversion rate from 14:0 to the new sn1 chain, providing normalized deacylation rates. For example, consider the value in the 14:1-14:1 experiment precursor experiment, from initial chain 14:1 to new sn1 chain 18:1. This value is 6.64. This value is obtained by looking at Supplementary Table 1 and calculating the ratio of the rate parameter for sn1 14:1 $\rightarrow$ 18:1 in the 14:1-14:1 precursor experiment and the rate parameter for sn1 14:0 $\rightarrow$ 18:1 in the 14:0-14:0 precursor experiment. This gives (0.5903/0.0889) = 6.64. After this normalization, we observe strong consistencies in each row, indicating robustly determined deacylation rates. Dashes indicate that the rate parameter was not in the dataset. Note that normalization of the rightmost column by the fastest rate (14:1) gives the relative values (0.15,1,0.46,-,0.12,0.03).

Experiment precursor	Initial chain	new sn1 chain			
		18:1	18:0	16:1	average
14:0-14:0	14:0	1	1	1	1
14:1-14:1	14:1	6.64	6.52	-	6.58 ± 0.08
18:3-18:3	18:3	2.81	3.92	2.26	3.00 ± 0.85
18:0-22:6	18:0	0.76	-	-	
18:0-18:1	18:0	0.79	-	-	0.78
18:1-18:1	18:1	-	0.11	0.28	0.20 ± 0.12

Table 3: sn1 relative reacylation rates. Rates in each row have been normalized by the conversion rate to 18:1 from the initial sn1 chain, providing normalized reacylation rates. After this normalization, we observe strong consistencies in each column, indicating robustly determined reacylation rates

Experiment precursor	Initial chain	new sn1 chain		
		18:1	18:0	16:1
14:0-14:0	14:0	1	0.19	0.29
14:1-14:1	14:1	1	0.18	-
18:3-18:3	18:3	1	0.26	0.24
average		1	0.21 ± 0.04	0.27 ± 0.04

Table 4: sn2 relative deacylation rates. Rates in each row have been normalized by the conversion rate from 14:0 to the new sn2 chain, providing normalized deacylation rates. After this normalization, we observe consistencies in each row, indicating robustly determined deacylation rates, though robustness is weaker than for the sn1 position.

Experiment precursor	Initial chain	new sn2 chain				
		18:1	18:2	16:1	20:4	average
14:0-14:0	14:0	3.7	1.52	-	-	2.61 ± 1.54
14:1-14:1	14:1	1	1	1	1	1
18:3-18:3	18:3	0.4	-	-	-	0.4
18:0-22:6	22:6	0.52	-	-	1.49	1 ± 0.69
18:1-18:1	18:1	-	0.02	-	-	0.02

Table 5: sn2 relative reacylation rates. Rates in each column have been normalized by the conversion rate to 18:1 from the initial sn2 chain, providing normalized reacylation rates. After this normalization, we observe qualitative consistencies in each column, though this effect is weaker than for the sn1 position

Experiment precursor	Initial chain	new sn1 chain			
		18:1	18:2	16:1	20:4
14:0-14:0	14:0	1	0.29	-	-
14:1-14:1	14:1	1	0.71	0.82	0.55
18:0-22:6	22:6	1	-	-	1.58
average		1	0.5 ± 0.3	0.82	1.07 ± 0.73

Table 6: Convergence of the fitting process. The initial guess is obtained by the shooting method. We then observe that there is a large improvement in fit in the first several steps of the fit procedure, though for the 18:0-18:1 case the initial guess is quite good. This behavior is obscured in Supplementary Figure 3 because of the large number of iterations and because the fit usually converges rapidly in the first few iterations. Note that for 18:0-18:1 and 18:1-18:1 there is no periodicity in the fitting process so we performed only a small number of iterations. The large change in error value in the initial few iterations indicates that the optimization is not heavily dependent on the initial guess. To further investigate this point, we also tested the effect of adjusting the initial guess algorithm, e.g. the number of splines used in the initial shooting. We found that optimized parameter values were not significantly impacted by the initial guess.

Experiment precursor	Number of Iterations (N)	Error at N	Error at N-4	Error at Initial Guess
14:0-14:0	1001	387.1	387.1	647.4
14:1-14:1	1005	1063.8	1070.6	2536.5
18:3-18:3	1004	2680.2	2680.1	3776.0
18:0-22:6	1009	752.8	752.9	1742.9
18:0-18:1	11	262.4	262.4 (N-1)	263.4
18:1-18:1	9	118.9	118.9 (N-1)	136.1

Table 7: Alternate solutions in the fitting process. For comparison, we analyzed other sets of parameter values found during the optimization process. Since the fits for the 14:0-14:0, 14:1-14:1, 18:3-18:3, and 18:0-22:6 experiments all exhibit 4-fold periodic behavior, for each of these experiments we considered the next best fit among the previous 3 iterations before the reported optimal solution. We found that these parameters had strong similarities to those in the reported optimal solution. Although some parameters, especially those with small absolute magnitudes, exhibited substantial variation, most parameters were consistent to within $\sim$ 40% of their value across fits. Even in cases with larger amounts of variation, the relative ordering of parameter values was often preserved. (top) Parameters in optimal solution (cf. Main text Table 2). (bottom) Parameters in next optimal solution.

**precursor**	**sn1**					**sn2**				
	Initial	*18:1*	*18:0*	*16:1*	*16:0*	Initial	*18:1*	*18:2*	*16:1*	*20:4*
$14:0-14:0$	*14:0*	0.0889	0.0166	0.0261	-	*14:0*	0.5176	0.1520	-	-
$14:1-14:1$	*14:1*	0.5903	0.1083	-	-	*14:1*	0.1400	0.0998	0.1153	0.0769
$18:3-18:3$	*18:3*	0.2500	0.0650	0.0589	-	*18:3*	0.0555	-	-	-
$18:0-22:6$	*18:0*	0.0673	-	-	-	*22:6*	0.0724	-	-	0.1146

**precursor**	**sn1**					**sn2**				
	Initial	*18:1*	*18:0*	*16:1*	*16:0*	Initial	*18:1*	*18:2*	*16:1*	*20:4*
$14:0-14:0$	*14:0*	0.0772	0.0093	0.0096	-	*14:0*	0.4735	0.2042	-	-
$14:1-14:1$	*14:1*	0.4469	0.1726	-	-	*14:1*	0.0597	0.0285	0.0378	0.0346
$18:3-18:3$	*18:3*	0.2499	0.0649	0.0589	-	*18:3*	0.0559	-	-	-
$18:0-22:6$	*18:0*	0.0673	-	-	-	*22:6*	0.0724	-	-	0.1145
